# Deficiency of mindin reduces renal injury after ischemia reperfusion

**DOI:** 10.1186/s10020-022-00578-2

**Published:** 2022-12-12

**Authors:** Tao Bai, Xiong Wang, Cong Qin, Kang Yang, Zhiguo Duan, Zhixiu Cao, Jiaqian Liang, Lei Wang, Jingdong Yuan, Pengcheng Luo

**Affiliations:** 1 Department of Urology, Wuhan No. 1 Hospital, Tongji Medical College, Huazhong University of Science and Technology, No. 215 Zhongshandadao, Qiaokou, Wuhan, 430022 China; 2 Department of Pharmacy, Tongren Hospital of Wuhan University (Wuhan Third Hospital), No. 241 Pengliuyang Road, Wuchang, Wuhan, 430060 China; 3 Department of Urology, QingPu Branch of Zhongshan Hospital Affiliated to Fudan University, No. 1158 Park Road (E), Qingpu, Shanghai, 201700 China; 4 Department of Urology, Renmin Hospital of Wuhan University, No. 238 Jiefang Road, Wuchang, Wuhan, 430060 China; 5 Department of Urology, Tongren Hospital of Wuhan University (Wuhan Third Hospital), No. 241 Pengliuyang Road, Wuchang, Wuhan, 430060 China

**Keywords:** Mindin, Acute renal injury, Renal ischemia reperfusion, Inflammation

## Abstract

**Background:**

Acute renal injury (AKI) secondary to ischemia reperfusion (IR) injury continues to be a significant perioperative problem and there is no effective treatment. Mindin belongs to the mindin/F-spondin family and involves in inflammation, proliferation, and cell apoptosis. Previous studies have explored the biological functions of mindin in liver and brain ischemic injury, but its role in AKI is unknown.

**Method:**

To investigate whether mindin has a pathogenic role, mindin knockout (KO) and wild-type (WT) mice were used to establish renal IR model. After 30 min of ischemia and 24 h of reperfusion, renal histology, serum creatinine, and inflammatory response were examined to assess kidney injury. In vitro, proinflammatory factors and inflammatory signaling pathways were measured in mindin overexpression or knockdown and vector cells after hypoxia/reoxygenation (HR).

**Results:**

Following IR, the kidney mindin level was increased in WT mice and deletion of mindin provided significant protection for mice against IR-induced renal injury as manifested by attenuated the elevation of serum creatinine and blood urea nitrogen along with less severity for histological alterations. Mindin deficiency significantly suppressed inflammatory cell infiltration, TNF-α and MCP-1 production following renal IR injury. Mechanistic studies revealed that mindin deficiency inhibits TLR4/JNK/NF-κB signaling activation. In vitro, the expression levels of TNF-α and MCP-1 were increased in mindin overexpression cells compared with vector cells following HR. Moreover, TLR4/JNK/NF-κB signaling activation was elevated in the mindin overexpression cells in response to HR stimulation while mindin knockdown inhibited the activation of TLR4/JNK/ NF-κB signaling after HR in vitro. Further study showed that mindin protein interacted directly with TLR4 protein. And more, mindin protein was confirmed to be expressed massively in renal tubule tissues of human hydronephrosis patients.

**Conclusion:**

These data demonstrate that mindin is a critical modulator of renal IR injury through regulating inflammatory responses. TLR4/JNK/NF-κB signaling most likely mediates the biological function of mindin in this model of renal ischemia.

## Introduction

Acute kidney injury (AKI) is a common clinical problem and a major cause of renal dysfunction, associated with high morbidity and mortality, often caused by insults such as kidney ischemia reperfusion (IR) injury, severe myocardial dysfunction, hypovolemia, sepsis, or nephrotoxins (Perico and Remuzzi 2015). It not only increases the personal economic burden, but is also closely related to prolonged hospital stay and poor clinical outcomes (Cooper and Fenves 2015). The estimated mortality rate of critically ill patients with AKI is 30–70% which are four times more likely to die in the hospital (Tian et al. 2014). Although we have made some progress in the study of pathogenesis and new therapeutic targets in recent years, physicians still do not have effective and specific treatment strategies.

Renal IR accounts for up to 60% of AKI cases and is well recognized as a significant cause of morbidity and mortality, especially in those admitted to the ICU (Basile et al. 2012). The pathophysiologic mechanism of renal IR injury include a complex interplay among acute cell injury, inflammation, and hemodynamic changes (Basile et al. 2012). In this process, inflammatory response play an important role in nephron destruction and renal dysfunction (Yang et al. 2016). Renal IR injury initiates an inflammatory cascade that exacerbates kidney damage, the infiltration of neutrophils and macrophages leads to the release of inflammatory cytokines and chemokines (Cen et al. 2016). These inflammatory mediators and cells induce renal cells death and tissue damage (Mizuno and Nakamura 2005). Therefore, inhibiting inflammation response is a promising strategy to protect the kidney from IR-mediated injury.

Mindin, a member of the mindin/F-spondin family, was first identified in zebra-fish as a component of the basal lamina (Feinstein and Klar 2004). Previous studies showed that mindin is involved in the regulation of neuron outgrowth (Feinstein and Klar 2004), infectious diseases (Jia et al. 2008), cardiac hypertrophy (Bian et al. 2012), neointima formation (Zhu et al. 2015), and metabolic diseases (Zhu et al. 2014). In addition, recently, mindin-deficient mice have been reported to be protected from IR mediated liver and brain injury (Sun et al. 2015; Wang et al. 2013). Although the role of mindin in the pathogenesis of diabetic nephropathy (DN) is well documented (Kahvecioglu et al. 2015; Murakoshi et al. 2011), mindin function in AKI is poorly understood.

In the present study, we tested the hypothesis that mindin is mechanistically linked to the pathogenesis of renal IR injury. In vivo, we studied the biological activities of mindin using wild-type and mindin knockout mice. In vitro, mindin stably overexpression cell line were generated and used to evaluate the influence of mindin on tubular epithelial cells (TECs) with injury induced by hypoxia/reoxygenation (HR). Here, we observed an up-regulation of mindin expression in kidney tissues after renal IR in mice and found that the loss of mindin prevents renal IR-induced injury by suppressing inflammatory responses. By contrast, high mindin promoted production of inflammatory cytokines and activation of inflammatory signaling in cultured TECs. And more, mindin protein interacts directly with TLR4 protein. These results show that in all probability, mindin is a critical mediator in renal IR injury, and a target gene with therapeutic potential.

## Materials and methods

### Animals and surgical protocols

All animal work were performed following the National Institutes of Health Guide for the Care and Use of Laboratory Animals and approved by the Animal Care and Use Committee of Renmin Hospital at Wuhan University. Male WT (C57BL/6) mice, mindin KO mice (on a C57BL/6 background; Stock NO. 02103) were obtained from the Center for Experimental Animals in Medicine College of Wuhan University (Hubei, China). All mice were maintained at constant temperature with alternating 12-h light/12-h dark cycle and fed with standard laboratory diet and ad libitum.

Male WT and mindin KO mice (8–10 weeks old, 20–25 g) were subjected to bilateral renal IR or a sham operation. Briefly, after the phenobarbital sodium (60 mg/Kg) was injected into the mice abdominal cavity, a heating pad was used to maintain the body temperature at 37 °C. Then, pedicels were exposed through a flank incisions and atraumatic microvascular clamps were used to interrupt the bilateral renal blood flow. The clamps were released after 30 min of ischemia, thereby initiating reperfusion. 24 h after reperfusion, all the mice from each group (n = 5) were sacrificed, then the serum and tissue samples of mice were collected. Sham animals underwent the same surgical procedure without arterial clamping.

### Human kidney tissues specimens

Human kidney tissue samples were obtained from five renal failure patients induced by hydronephrosis, which underwent surgical resection of their kidney (at Tongren Hospital of Wuhan University (Wuhan Third Hospital)). The study was approved by the ethical committee of Tongren hospital of Wuhan University (the third hospital of Wuhan). Informed consent was obtained from all the patients. Additionally, this study has been conducted according to the Code of Ethics of the World Medical Association (Declaration of Helsinki) for experiments involving humans.

### Cell culture and treatment

Human renal tubular epithelial cells (HK-2) were obtained from China Center for Type Culture Collection (Hubei, China). Mindin-stably overexpression HK-2 cells (admindin) and control cells (adGFP), Mindin, TLR4, JNK knockdown HK-2 cells and control cells (siRNA) were established. Mindin (accession No. NM_001199021), TLR4 (accession No. NM_003266) and JNK (accession No. NM_001278548) siRNA were provided by Wuhan Yingji Technology Co., Ltd (Wuhan, China). The gene sequence were designed as follows: si-Mindin-F: CGCUGAUGAAGGAGAUAUCGATT, si-Mindin-R: UCGAUCUCCUUCAUCAGCGTT, si-TLR4-F: CAUUGGAUACGUUUCCUUATT, si-TLR4-R: UAAGGAAACGUAUCCAAUGTT, si-JNK-F: GCUGGUAAUAGAUGCAUCUTT, si-JNK-R: AGAUGCAUCUAUUACCAGCTT. These cells were maintained in MEM media that contained 10% fetal bovine serum (FBS) at 37 °C under normoxic conditions (95% air/ 5% CO_2_). HR model was used to mimic renal IR injury in vitro. After cultured in serum-free MEM medium for 24 h, cells were replaced with glucose-free MEM medium, and incubated under hypoxia atmosphere (containing 5% CO_2_, 1.0% O_2_, and 94% N_2_) at 37 °C for 24 h. Then, the cells were cultured in complete medium under normoxic conditions (95% air/5% CO_2_) for the indicated times.

### Establishment of a stable cell line

After double enzyme digestion of pLVX-3FLAG-mCMV-ZsGreen-IRES-Puro and pUC57-hSPON2 (Huameng BioTech, Wuhan) by SpeI and BamHI (NEB), the fragments were connected by T4 DNA Ligase (M0202L, NEB). Then, the recombinant plasmids were transformed into JM109 (Huameng BioTech, Wuhan) and transformants were selected on LB agar plates containing ampicillin (100 μg/ml). A Small Plasmid Extraction Kit (EM101, Transgen BioTech, Beijing) was used to extract plasmids. By restriction enzyme identification and sequence analysis, the recombinant expression plasmid was successfully constructed. Then, the HEK293T cells were co-transfected with the recombinant plasmids (pLVX- hSPON2-ZsGreen-Puro, pLVX-ZsGreen-Puro) to generate high titer lentiviral particles (rLV-hSPON2-ZsGreen-Puro, rLV-ZsGreen-Puro). Next, HK-2 cell lines were infected with rLV-hSPON2-ZsGreen-Puro and rLV-ZsGreen-Puro on the basis of MOI = 50, and then selected with 5 µg/mL puromycin in complete culture medium. The mindin gene overexpression cloned information is as followed: Gene name Mindin: accession No. NM_012445.3, Cloning Vector: pLVX-3FLAG-mCMV-ZsGreen-IRES-Puro, Cloning Strategy: SpeI + BamHI, Mindin synthesis sequence: 1008 bp. Admindin (mindin-overexpression group) and adGFP cells (empty vector group) were obtained.

### Cell viability assay

Mindin-stably overexpression HK-2 cells (admindin) and control cells (adGFP), Mindin, TLR4, JNK knockdown HK-2 cells and control cells (siRNA) were established respectively. And more, different concentration of IL-1β inhibitor YQ128 (MCE, Cat No. HY-130252) (0.1, 0.2, 0.5, 1.0, 2.0, 5.0, 10.0, 20.0 μM) were added into each well in a 96-well plate. Cell viability was measured using the Cell Counting Kit-8 (CCK-8) assay (Biosharp, Beijing Labgic Technology Co., Ltd). According to the manufacturer’s instructions, log-phase HK-2 cells were collected and adjusted to 1 × 10^5^ cells/ml. A 100 μL aliquot of the cell suspension was added to each well in a 96-well plate. Ten microlitres of the CCK-8 stock solution was added to the media. The optical density values (OD values) were measured at 450 nm by absorbance microplate reader at 0, 24, 48 and 72 h (CMAX PLUS, Molecular Devices). The cell viability and inhibition ratios were calculated by OD values.

### Renal function assay

Blood samples were collected when mice were sacrificed, then samples were centrifuged at 4 °C for 10 min to separate plasma. Renal function was monitored by measuring the serum creatinine (Cr) and blood urea nitrogen (BUN) concentration using a fully automatic biochemical detector at Renmin Hospital of Wuhan University.

### Hematoxylin eosin (HE) assay

To assess renal tubular injury and the inflammatory status, kidney tissue samples were fixed with 4% formaldehyde for 24 h, embedded in paraffin. After deparaffinized, 5 μm sections were stained with HE. Histological changes in the cortico-medullary region were examined under an Olympus microscope (Olympus, Tokyo, Japan) and kidney injury was evaluated by using the acute tubular necrosis (ATN) score. Briefly, tubular injury was estimated in 5 random high-power fields (HPF; 400) per section by using a scoring system based on the percentage of damaged tubules per field (0, none; 1, < 11%; 2, 11% to 25%; 3, 26% to 45%; 4, 46% to 75%; 5, > 75%). For assessment of renal injury, includes tubular cell necrosis, tubular distension, cast formation, and interstitial widening ^17^. The 5 μm paraffin sections were evaluated in a blind manner by a pathologist. To guarantee random selection, the fields for each sample were selected by a freshman from the Renmin hospital who was able to operate the microscope.

### Immunohistochemistry (IHC) assay

Human or mice kidney tissues were fixed in 4% paraformaldehyde, embedded in paraffin and sectioned into 5 μm thick slices, mounted on glass slides. Tissue sections were then deparaffinized and rehydrated, antigen was exposed by heating in a microwave oven to 100 °C in sodium citrate for 15 min. Then, the sections were incubated in 0.3% hydrogen peroxide for 20 min to blocked endogenous peroxidase activity. After incubated with 10% goat serum for 30 min at room temperature, the kidney sections were incubated overnight at 4 °C with the following primary antibodies against mindin (1:50; sc166868; Santa Cruz), LY6G (1:1000; ab238132; Abcam), CD68 (1:100; ab125047; Abcam), TNF-α (1:100; ab6671; Abcam), and MCP-1 (1:100; sc28879; Santa Cruz). Subsequently, the sections were rinsed and incubated with horseradish peroxidase (HRP)-conjugated anti-rabbit/mouse IgG secondary antibodies (Dako, Glostrup, Denmark) for 25 min at room temperature. Immune reaction products were developed with DAB. We randomly selected 10 fields/sample to photograph images under a microscope at 400 × magnification and measure positive signals using ImageJ 1.52a software. The data were then averaged to calculate the positive area for each sample.

### Quantitative real-time PCR analysis

After HK-2 cells were treated according to the experimental design, the cells were dissolved in Trizol reagents. Total RNA was extracted with the RNeasy Plus Mini RNA extraction kit in line with the manufacturer instructions. Then the extracted RNA concentration was determined using a spectrophotometer (Quawell Q3000, USA) at 260 nm wavelength. The RNA purity was determined by assaying the A260/A280 ratio between 1.8 and 2.1. The strand complimentary DNA was synthesised from RNA via reverse transcriptase and a PrimeScript RT reagent kit (Thermo Fisher, USA). Quantitative real-time PCR was carried out in a Real-Time PCR system (ABI7500, USA). Each reaction was run in triplicate and performed in 35 cycles consisting of the following steps: initial denaturation at 94 °C for 10 min followed by a set cycle of denaturation at 94 °C for 15 s and different annealing temperatures for each pair of primers (ranging between 53 and 62 °C) for 10 s, extension at 72 °C for 28 s and a final elongation at 72 °C for 5 min. At last, a melt curve analysis was performed. Primer sequences of the targeted genes used in this study were as follows: TLR4 (5ʹ-AGACCTGTCCCTGAACCCTAT-3ʹ, forward; 5ʹ-CGATGGACTTCTAAACCAGCCA-3ʹ, reverse), JNK (5ʹ-TGTGTGGAATCAAGCACCTTC-3ʹ, forward; 5ʹ-AGGCGTCATCATAAAACTCGTTC-3ʹ,reverse), NF-κB p65 (5ʹ-AACAGAGAGGATTTCGTTTCCG-3ʹ, forward; 5ʹ-TTTGACCTGAGGGTAAGACTTCT-3ʹ, reverse), MCP1 (5ʹ-CAGCCAGATGCAATCAATGCC-3ʹ, forward; 5ʹ-TGGAATCCTGAACCCACTTCT-3ʹ, reverse) and β-actin (5ʹ-CATGTACGTTGCTATCCAGGC-3ʹ, forward; 5ʹ-CTCCTTAATGTCACGCACGAT-3ʹ, reverse). Specific PCR products subjected to melting curve analysis for each primer set revealed only one peak for each product. Every gene expression level was normalised for β-actin expression, and represented as the fold ratio compared with control group respectively.

### Western blot assay

Renal sample or cultured cells were homogenized and lysed in RIPA buffer, and bicinchoninic acid reagen was used to determine protein concentration. Equal amounts of protein were resolved onto SDS-PAGE gel, and transferred onto a PVDF membrane (Millipore, MA, USA). The blot was blocked in 5% bovine serum albumin for 1 h, then, incubated overnight at 4 °C with following primary antibodies against mindin (1:1000; ab171955; Abcam), TLR4 (1:500, ab13556; Abcam), phospho-JNK (1:1000; 4668; Cell Signaling Technology), JNK (1:1000; 9252; Cell Signaling Technology), phospho-NF-κB p65 (1:1000; 3033; Cell Signaling Technology), NF-κB p65 (1:1000; 4814; Cell Signaling Technology), phospho-IκBα (1:1000; 2859; Cell Signaling Technology), IκBα (1:1000; 4814; Cell Signaling Technology), and GAPDH (1:10,000; ab181602; Abcam). Washed three times with TBST, the blots were incubated with secondary anti-mouse antibody (1:15,000; 5257; Cell Signaling Technology) or anti-rabbit antibody (1:15,000; 926-32211; LI-COR) for 1 h. Bands were scanned and visualized using an Odyssey Infrared Imaging system (LI-COR, USA) with Odyssey Application Software (version 3.0, LI-COR Biosciences, USA).

### Immunofluorescence assay

For cultured cell fixation, cells were fixed in 4% paraformaldehyde. Subsequently, permeabilized with 0.1% Triton X-100 (Biosharp, Hubei, China) for 5 min, and blocking with BSA for 1 h. Then the cells were stained with primary antibody targeting mindin (1:50; sc-166868; Santa Cruz) for 2 h and Alexa Fluor 488 conjugated donkey anti-mouse IgG secondary antibody (1:400; A21202; Invitrogen) for 1 h. The nuclei were stained with DAPI. After washes, slides were mounted with the antifade reagent.

### Enzyme-linked immunosorbent assay (ELISA)

The serum cytokine levels of TNF-α and MCP-1 were determined by using specific commercially available ELISA kits (TNF-α catalogue number PT512, Beyotime Biotechnology, China; MCP-1 catalogue number PC125, Beyotime Biotechnology, China) according to the manufacturer’s instructions.

### Co-immunoprecipitation (Co-IP) assays

The HK-2 cells were lysed in immunoprecipitation lysis buffer with protease inhibitor (bioswamp, Cat. No. PAB180006). Then the cellular lysate supernatant was obtained by centrifugation (10,000 × *g*, 30 min, 4℃). Later the protein concentration was assayed by the BCA kit (bioswamp, Cat. No. PAB180007). For immunoprecipitation of mindin, the cell samples containing 500 μg of total protein were pre-cleaned via incubition with protein A/G magnetic beads (MCE, Cat. No. HY-K0204) for 30 min at 4℃. The supernatant was incubated with anti-mindin mouse monocloning antibody (1:1000, Santa Cruz) at 4 ℃ overnight. The acquired immune complexes were precipitated by protein A/G agarose beads at 4 ℃ for 2 h. The immune-precipitates were washed 5 × with lysis buffer on ice and re-suspended with Laemmli sample buffer. Then the samples were analysed by SDS-PAGE and immunodetected using anti-TLR4 antibody (1:1000, Proteintech Group, Inc, China, 66350-1-Ig), and checked by a mouse anti-mindin antibody(Santa Cruz, USA, sc-166868). Furthermore, the whole cell lysates were performed to measure mindin and TLR4 proteins by immunoblotting.

### Surface plasmon resonance technology (SPR) assays

The SPR test was performed to demonstrate the binding of mindin to TLR4 proteins. Mindin protein (HY-P70142, MedChemExpress) and TLR4 protein (HY-P73586, MedChemExpress) were used to study the protein binding in CM5 electronic chips by biomolecular interaction instrument (Biacore T2000). Different concentrations of TLR4 protein (3.125, 6.25, 12.5, 25.0, 50.0,100.0 nM) were carried out to analyse the binding and dissociation rate constants between mindin and TLR4 proteins. At last, the binding constant of them was calculated to evaluate protein binding.

### Statistical analysis

Data in this study represent mean ± standard deviation. One-way analysis of variance was used to compare data and calculate P value. All experiments were repeated at least three times. Data analyses were done with the software SPSS 23.0 for Windows (IBM, Armonk, NY, USA). P-values less than 0.05 was regarded significant.

## Result

### Mindin expression is elevated in renal IR injury in vivo and in vitro

**Fig. 1:**
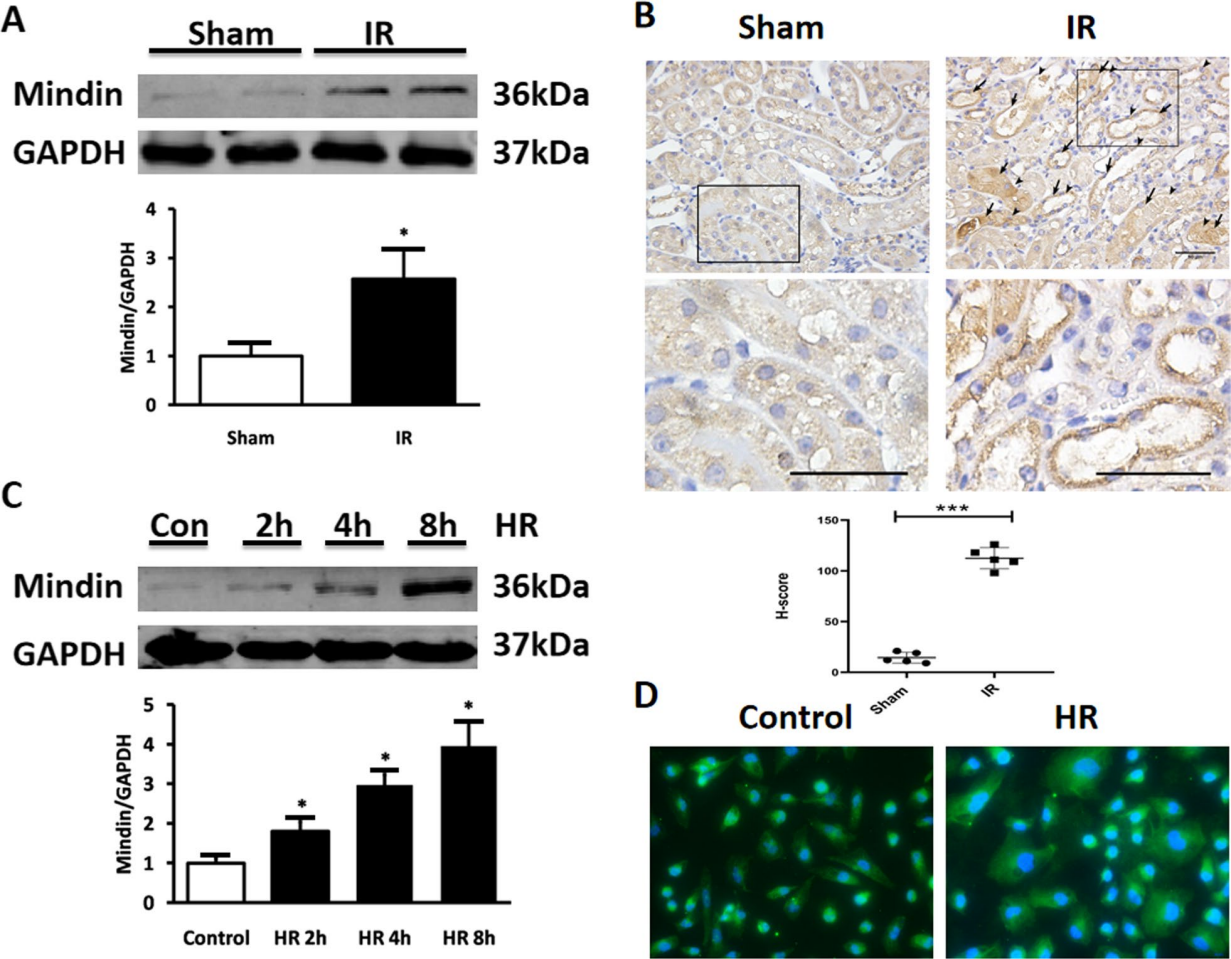
Mindin expression was elevated in renal IR injury in vivo and in vitro. **A** Mindin measured by western blot analysis in sham mice and IR mice. Quantitative analysis of mindn protein expression. Expression levels were normalized to GAPDH and are presented relative to the sham-operated kidney. *P < 0.05 versus Sham. **B** Representative photomicrographs showing IHC analysis for mindin after 30 min renal ischemia and 24 h reperfusion (magnification 400 ×). **C** Mindin measured by western blot analysis in HK-2 cells after 24 h hypoxia and 2, 4, and 8 h reoxygenation. Quantitative analysis of mindin protein expression. Expression levels were normalized to GAPDH and are presented relative to the control cells. *P < 0.05 versus Control. **D** Representative photomicrographs showing immunofluorescence staining for mindin after 24 h hypoxia and 8 h reoxygenation (magnification 400 ×)

To explore the potential role of mindin in AKI, we first examined expression change of mindin in WT mice subjected to bilateral renal arterial occlusion followed by a period of reperfusion. We observed that the expression of mindin was upregulated after 30 min ischemia and 24 h reperfusion (Fig. 1A). Furthermore, IHC results demonstrated that mindin expression was significantly increased in tubules after IR (Fig. 1B, Arrowheads indicate injured tubules; arrows indicate mindin staining).

In accordance with the in vivo results, mindin expression was also elevated after 2 h reoxygenation and significantly increased about three times after 8 h reoxygenation in HK-2 cells compared with untreated cells (Fig. 1C). Meanwhile, immunofluorescence analyses of cultured cells were also confirmed this upregulation (Fig. 1D). The induced mindin expression in kidney implied that mindin might be critical in renal IR injury.

### Mindin deficiency attenuates renal IR injury

**Fig. 2:**
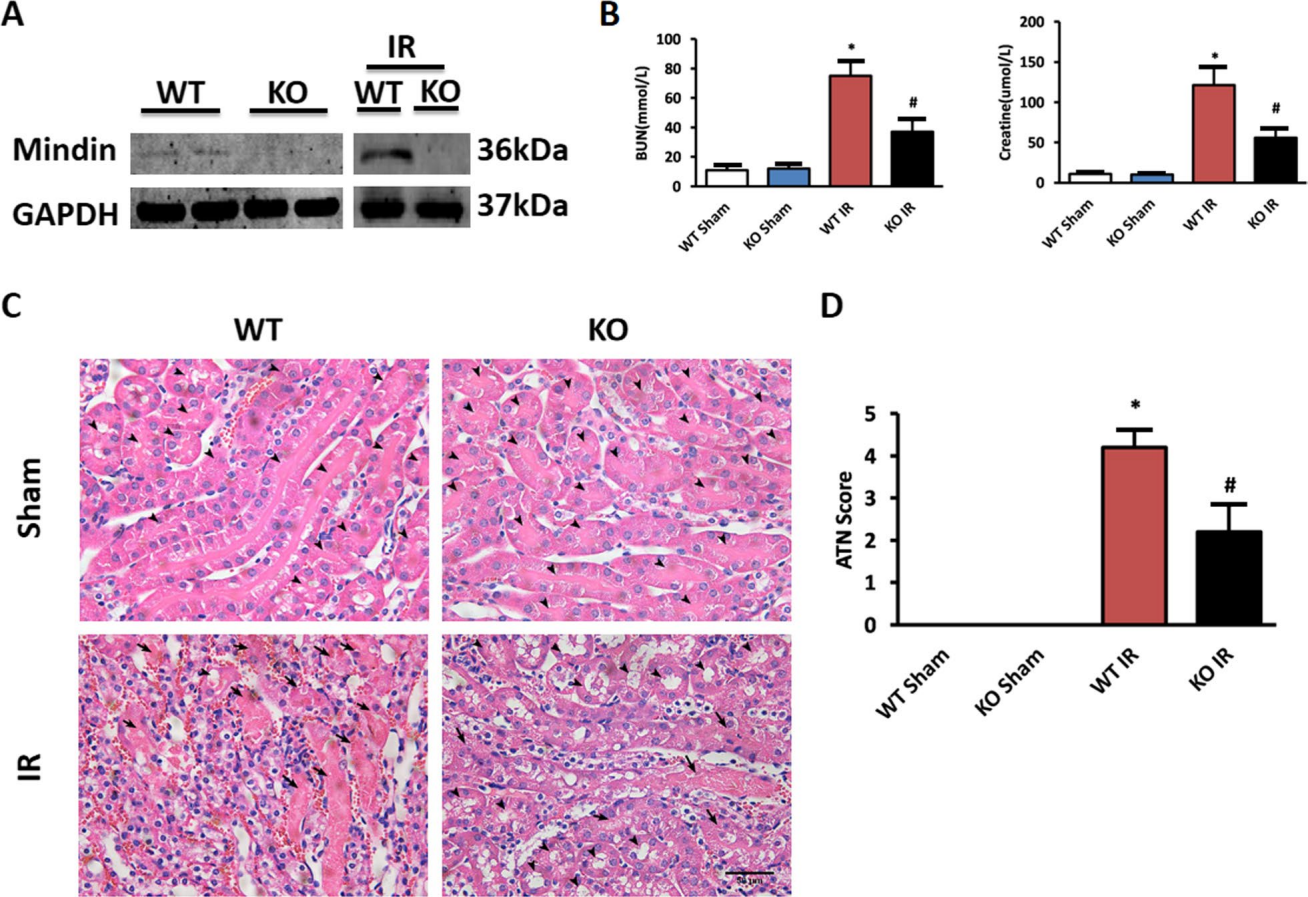
Mindin deficiency attenuated renal IR injury. **A** Mindin protein expression in kidneys from WT and Mindin KO mice with and without IR. **B** Levels of BUN and Cre after renal IR injury in WT and mindin KO mice. *P < 0.05 versus WT Sham or KO Sham. ^#^P < 0.05 versus WT IR. **C** Representative photomicrographs showing HE staining in WT and mindin KO mice with or without renal IR injury (magnification 400 ×). **D** Degree of renal damage graded using the acute tubular necrosis (ATN) score. *P < 0.05 versus WT Sham or KO Sham. ^#^P < 0.05 versus WT IR

The upregulation of mindin in kidney prompted us to investigate the role of mindin during the progression of IR-induced renal injury. To address this, WT and mindin KO mice were used to established renal IR model. At first, mindin ablation was confirmed in the kidney of KO mice by western blot analyses (Fig. 2A). Next, we evaluated the levels of serum blood urea nitrogen (BUN) and creatinine (Cre), markers of renal dysfunction. In WT mice, serum BUN and Cre concentration both increased by 80% after IR surgery, while these parameters markedly decreased by 50% in mindin KO mice compared with WT mice (Fig. 2B). In addition, histologic examination showed extensive tubular injury characterized by tubular cell death, dilation of tubules, loss of brush border and cast formation in the outer medulla region in WT mice after IR (Arrowheads indicate brush border; arrows indicate intraluminal debris). However, kidney specimens from mindin KO mice demonstrated less tubular injury (Fig. 2C). More precisely, we assessed overall tissue injury by ATN score and we confirmed the mitigated tissue injury in mindin KO mice after renal IR, as compared to WT mice (Fig. 2D).

### Mindin deficiency limits renal IR induced inflammatory cell infiltration and proinflammatory mediators production

**Fig. 3:**
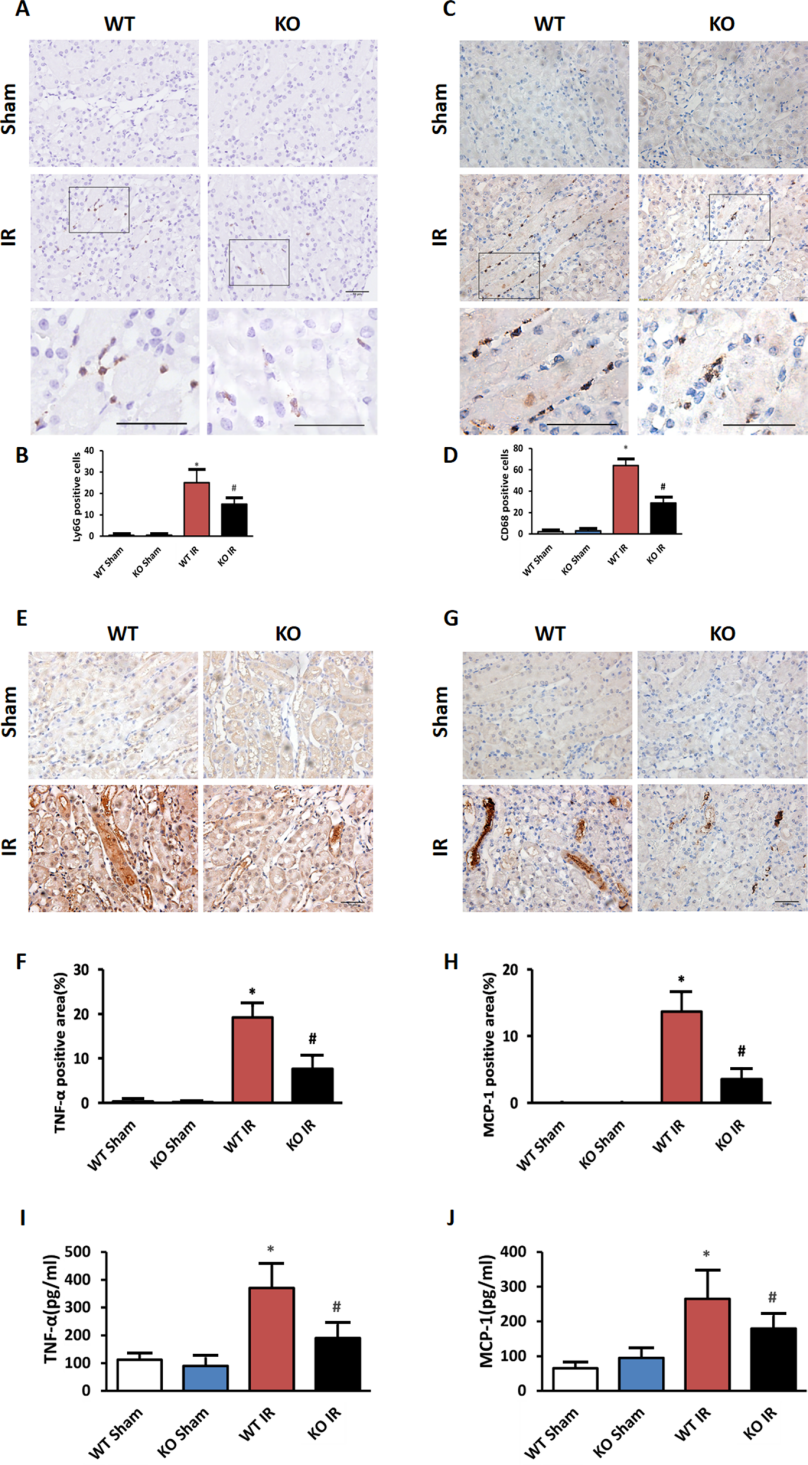
Mindin deficiency limited renal IR induced inflammatory cell infiltration and proinflammatory mediators production. **A** Representative photomicrographs showing IHC staining of LY6G in WT and mindin KO mice induced by IR or not (magnification 400 ×). **B** Quantitative analysis of LY6G in the indicated groups. *P < 0.05 versus WT Sham or KO Sham. ^#^P < 0.05 versus WT IR. **C** Representative photomicrographs showing the IHC staining of CD68 in WT and mindin KO mice induced by IR or not (magnification 400 ×). **D** Quantitative analysis of CD68 in the indicated groups.*P < 0.05 versus WT Sham or KO Sham. ^#^P < 0.05 versus WT IR. **E** Representative photomicrographs showing IHC staining of TNF-α in WT and mindin KO mice induced by IR or not (magnification 400 ×). **F** Quantitative analysis of TNF-α in the indicated groups.*P < 0.05 versus WT Sham or KO Sham. ^#^P < 0.05 versus WT IR. **G** Representative photomicrographs showing IHC staining of MCP-1 in WT and mindin KO mice induced by IR or not (magnification 400 ×). **H** Quantitative analysis of MCP-1 in the indicated groups. *P < 0.05 versus WT Sham or KO Sham. ^#^P < 0.05 versus WT IR. **I**, **J** Quantitation of TNF-α and MCP-1 in serum was measured by ELISA. *P < 0.05 versus WT Sham or KO Sham. ^#^P < 0.05 versus WT IR

Given that inflammatory responses play an important role in IR-induced AKI (Kono et al. 2013), we wondered whether mindin deficiency could ameliorate renal IR injury by regulating the inflammatory response. HE staining was performed to evaluate the inflammatory status of kidney after renal IR. As shown in Fig. 2C, compared with WT mice, mindin deficiency markedly attenuated inflammatory cell infiltration after renal IR injury. In order to further confirm the effects of regulating the recruitment of inflammatory cells, we examined neutrophils and macrophage accumulation by immunostaining of LY6G and CD68, respectively. As shown in Fig. 3A–D, prominent neutrophils and macrophages infiltration was noted in WT mice after IR insult, however, ablation of mindin suppressed inflammatory cells infiltration significantly (about 50%). In response to acute insult, TECs and activated leukocytes release inflammatory mediators, and these cytokines and chemokines act in concert to promote inflammation in a positive feedback loop promoting further kidney damage. Therefore, we next examined the production of TNF-α and MCP-1 in kidney specimens following renal IR injury in WT and KO mice. IHC staining revealed that the local production of these proinflammatory molecules were markedly increased after renal IR injury in WT mice, but this effect was reduced about 50% by mindin deletion (Fig. 3E–H). We also determined the serum circulation levels of TNF-α and MCP-1 following renal IR injury in WT and KO mice. Consistent with the IHC results, the production of proinflammatory was decreased in mindin KO mice after renal IR injury compared with WT mice (Fig. 3I, J). Collectively, these combined data indicated that mindin ablation provides a protective effect against renal inflammatory cell infiltration and inflammatory mediators production induced by IR injury.

### Mindin deficiency inhibits TLR4/JNK/NF-κB signaling activation in renal IR injury

**Fig. 4:**
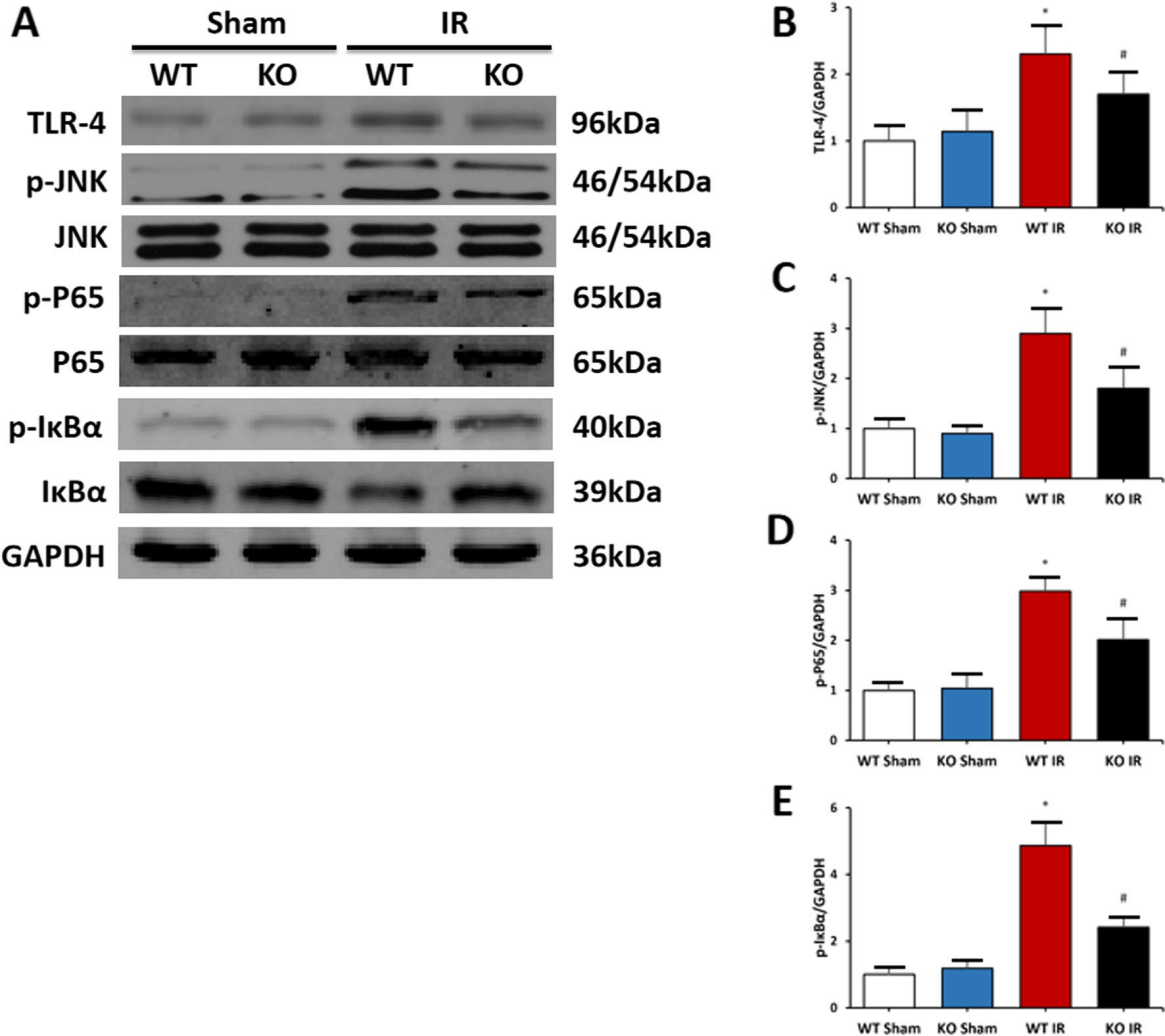
Mindin deficiency inhibited TLR4/JNK/NF-κB signaling activation in renal IR injury **A** TLR4, phosphorylated JNK, phosphorylated P65, and phosphorylated IκBα measured by western blot analysis in kidneys of WT and mindin KO mice induced by IR or not. **B**–**E** Quantitative analysis of TLR4, phosphorylated JNK, phosphorylated P65, and phosphorylated IκBα protein expression. *P < 0.05 versus WT Sham or KO Sham. ^#^P < 0.05 versus WT IR

Toll-like receptor 4 (TLR4), one of the members of the Toll-like receptor family, has been found to be involved in a variety of diseases associated with inflammation, such as renal IR injury. Moreover, the JNK/NF-κB signaling was associated closely with the activation of TLR4 and contributed to the production of inflammatory mediators, such as MCP-1 and TNF-α (Song et al. 2013). Therefore, having confirmed that loss of mindin mitigates inflammatory responses, we wished to determine whether mindin ablation suppressed the TLR4/JNK/NF-κB signaling activation during renal IR injury. We measured the expression levels of TLR4/JNK/NF-κB/ signaling using western blot analysis. As shown in Fig. 4A–E, TLR4, phospho-JNK, phospho-P65, and phospho-IκBα expression levels were upregulated by 60% in the kidneys with AKI, while ablation of mindin suppressed the activation of TLR-4/JNK/NF-κB signaling after IR injury as revealed by about 40% decreased expression levels of TLR4, phospho-JNK, phospho-P65, and phospho-IκBα.

### Mindin overexpression promotes production of inflammatory mediators and activation of TLR4/JNK/NF-κB signaling after HR in vitro

**Fig. 5:**
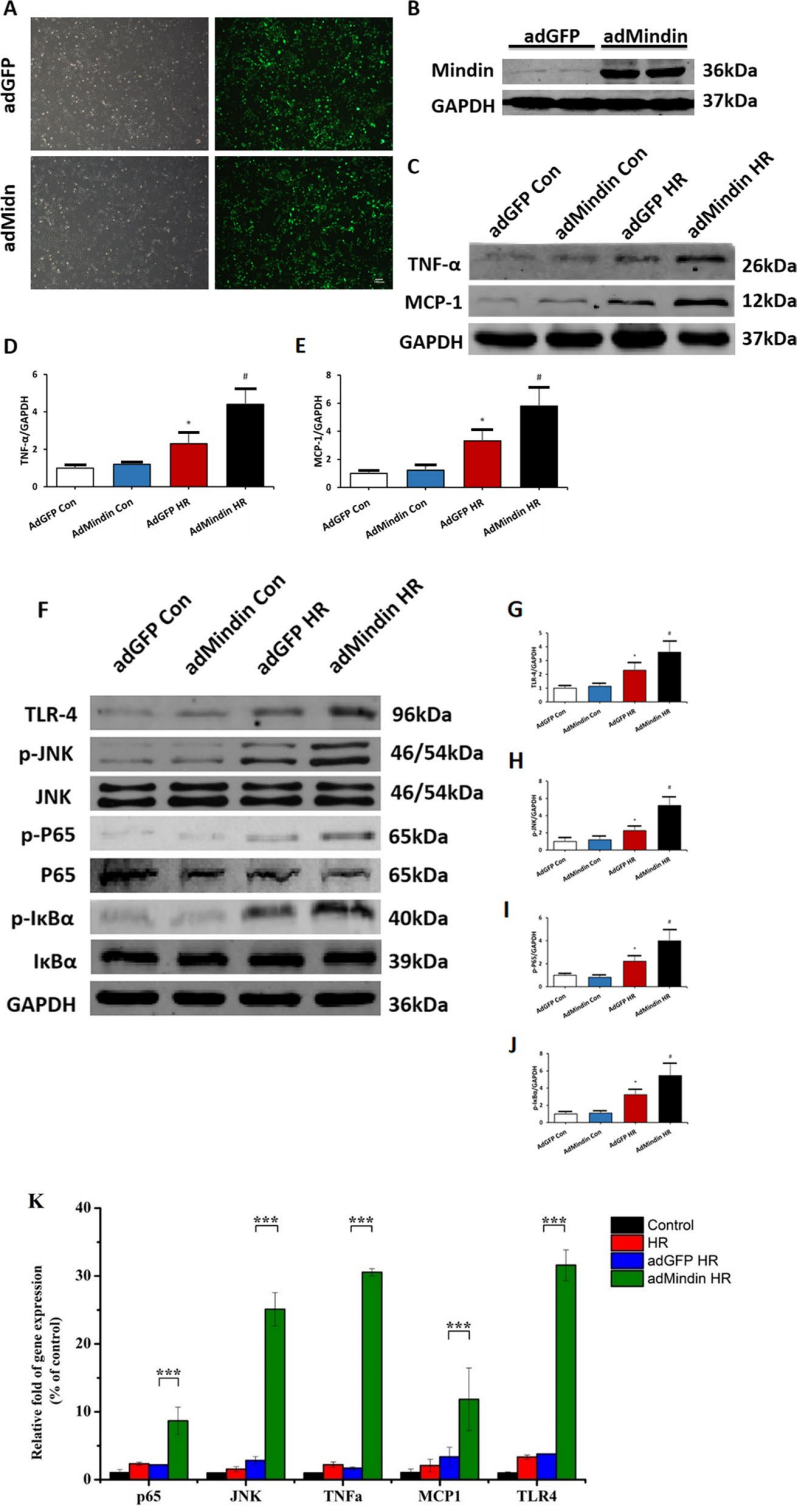
Mindin overexpression promoted production of inflammatory mediators and activation of TLR4/JNK/ NF-κB signaling after HR in vitro. **A** Representative photomicrographs showing the immunofluorescence of GFP in adGFP and adMindin cells. **B** Mindin protein expression in adGFP and adMindin cells. **C**–**E** Protein levels of TNF-α and MCP-1 in the media of cultured TECs by Western blot. *P < 0.05 versus adGFP Con or adMindn Con. ^#^P < 0.05 versus adGFP HR. **F** TLR4, phosphorylated JNK, phosphorylated P65, and phosphorylated IκBα measured by western blot analysis in adGFP and adMindin cells induced by HR or not. **G**–**J** Quantitative analysis of TLR4, phosphorylated JNK, phosphorylated P65, and phosphorylated IκBα protein expression. *P < 0.05 versus adGFP Con or adMindn Con. ^#^P < 0.05 versus adGFP HR. **K** Quantitative analysis of TLR4, JNK, P65, TNF-α and MCP-1 mRNA expression in mindin-overexpressed HK-2 cells. ***P < 0.001 versus adGFP HR group

The beneficial effect of mindin deficiency on inflammatory response during renal IR was confirmed in vivo, next, to further test the direct contribution of mindin to release of inflammatory factors and activation of TLR4/JNK/ NF-κB signaling in vitro, we established mindin stably transfected HK-2 cells (adMindin) and control cells (adGFP) (Fig. 5A, B). AdMindin cells and adGFP cells were challenged by HR, the expression levels of inflammatory factors and TLR4/JNK/NF-κB signaling in response to HR stimulation were examined by western blot analysis, respectively. The results revealed that TNF-α and MCP-1 protein expression levels of cultured TECs were significantly increased following HR. Moreover, mindin overexpression cells showed substantially higher TNF-α and MCP-1 levels when compared with the adGFP cells after HR (Fig. 5C–E). In addition, compared to adGFP group, high expression of mindin significantly increased the activation of TLR4/JNK/NF-κB signaling in response to HR stimulation (Fig. 5F–J). Except protein level, mRNA quantitative analysis also showed that TLR4, JNK, P65, TNF-α and MCP-1 expression were increased significantly in mindin-overexpressed HK-2 cells (Fig. 5K). These data reinforces the possibility mindin could be critical in renal IR-mediated inflammatory response, and mindin may be a potential target gene for the treatment of ischemic AKI.

### Mindin knockdown inhibited the activation of TLR4/JNK/ NF-κB signaling after HR in vitro

**Fig. 6:**
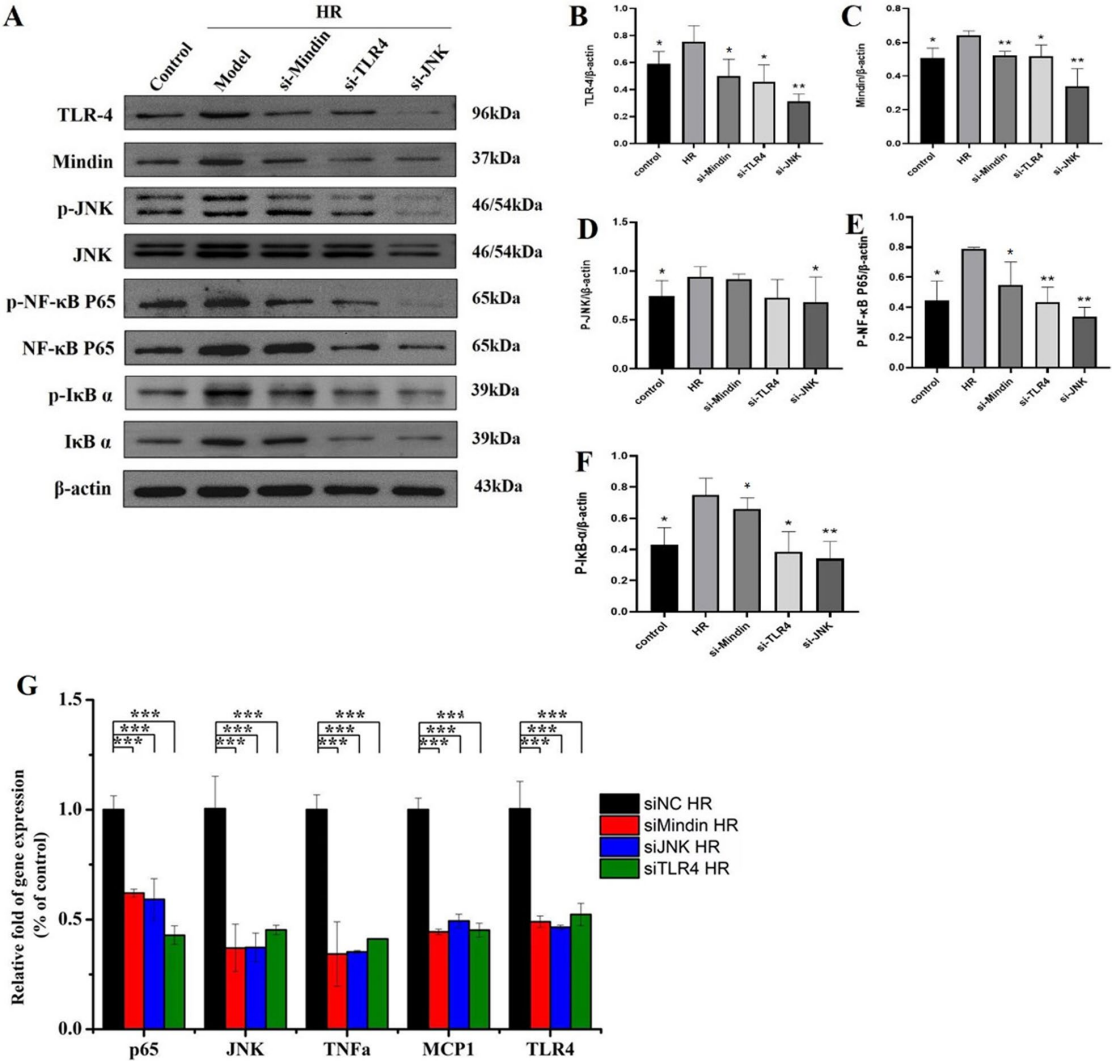
Mindin knockdown inhibited the activation of TLR4/JNK/ NF-κB signaling after HR in vitro. **A** TLR4, Mindin, phosphorylated JNK, phosphorylated P65, and phosphorylated IκBα measured by western blot analysis in si-Mindin, si-TLR4 and si-JNK cells induced by HR respectively. **B**–**F** Quantitative analysis of TLR4, Mindin, phosphorylated JNK, phosphorylated P65, and phosphorylated IκBα protein expression. ^*^P < 0.05, ^**^P < 0.01 versus HR. **G** Quantitative analysis of TLR4, JNK, P65, TNF-α and MCP-1 mRNA expression in si-Mindin, si-TLR4 and si-JNK cells induced by HR respectively. ***P < 0.001 versus control HR group

To further test the direct effect of mindin to the activation of TLR4/JNK/ NF-κB signaling in vitro, we established mindin, TLR4 or JNK knockdown HK-2 cells and control cells(siRNA) (Fig. 6A). Treated by HR, the expression levels of inflammatory factors and TLR4/JNK/NF-κB signaling in response to HR stimulation were examined by western blot and mRNA quantitative analysis respectively. The results showed that compared to HR group, low expression of mindin inhibited the activation of TLR4/JNK/NF-κB signaling in response to HR stimulation significantly (Fig. 6B–G).

### Mindin inhibited cell proliferation significantly

Treated by HR, the effect of mindin overexpression or knockdown on cell proliferation was observed in HK-2 cell at 0, 24, 48 and 72 h.

**Fig. 7:**
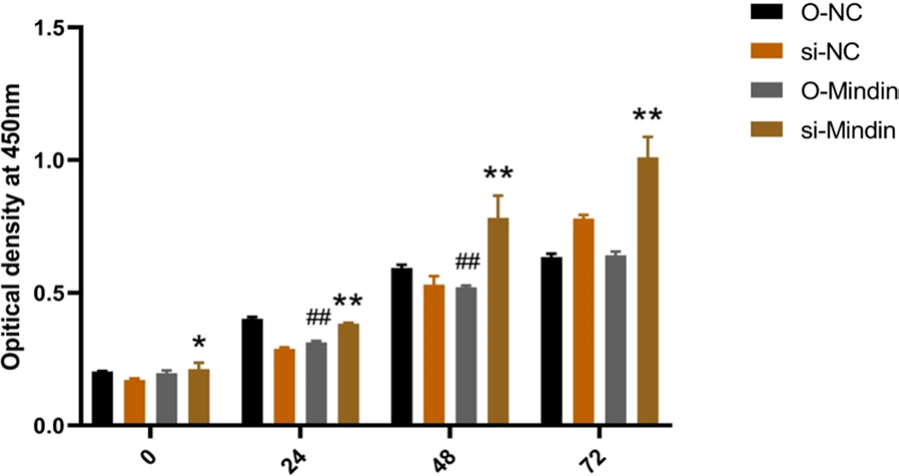
Mindin inhibited cell proliferation significantly. The effect of Mindin overexpression or knockdown on cell proliferation was studied in HK-2 cell. O-NC: adGFP normal control group, si-NC: siRNA normal control group, O-Mindin: Mindin overexpression group, si-Mindin: Mindin siRNA knockdown group. ^*^P < 0.05, ^**^P < 0.01 versus si-NC group; ^#^*P* < 0.05, ^##^*P* < 0.01 versus O-NC group. Interaction between the time and gene interference factors, *F* = 49.448, *P* < 0.001; main effect of the time factor, *F* = 1298.047, *P* < 0.001

Compared with siRNA control (si-NC) group, Mindin knockdown(siRNA) increased cell viability at 24 h (*P* < 0.01), 48 h (*P* < 0.01) and 72 h (*P* < 0.01) respectively. However, compared with adGFP normal control(O-NC) group, Mindin overexpression inhibited cell viability at 24 h (*P* < 0.01) and 48 h (*P* < 0.01) significantly (Fig. 7).

### Inhibition of IL-1β attenuated cell proliferation

**Fig. 8:**
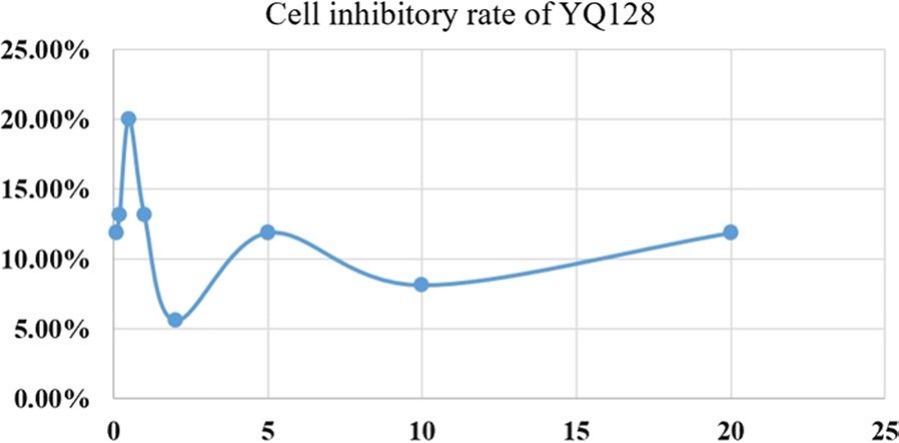
Inhibition of IL-1β attenuated cell proliferation significantly. Treated by various concentration of YQ128 (0.1, 0.2, 0.5, 1.0, 2.0, 5.0, 10.0, 20.0 μM) in HK-2 cell respectively, the cell viability was measured using the CCK-8 assay. Compared with control group, the maximum cell inhibitory rate reached up to 20% treated by 0.5 μM dose of YQ128

Treated by various concentration of YQ128 (0.1, 0.2, 0.5, 1.0, 2.0, 5.0, 10.0, 20.0 μM) in HK-2 cell respectively, cell viability was measured using the CCK-8 assay. Compared with control group, the maximum cell inhibitory rate reached up to 20% treated by 0.5 μM dose of YQ128 (Fig. 8).

### Mindin protein interact directly with TLR4 protein

**Fig. 9:**
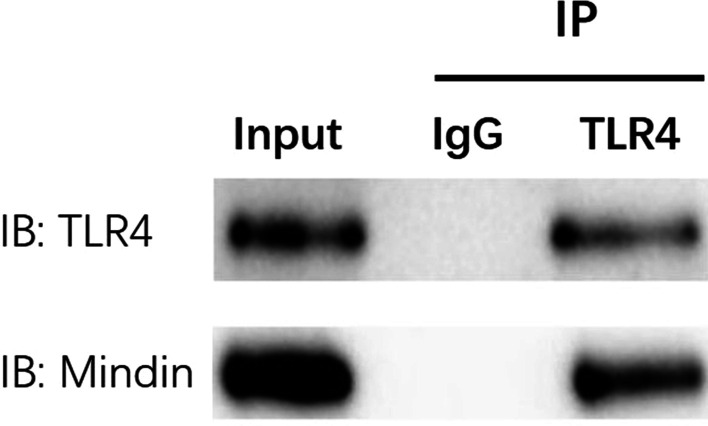
Co-immunoprecipitation of TLR4 and mindin in HK-2 cells. Lysates of HK-2 cells were incubated with anti-TLR4 antibody and immune complexes were precipitated by protein A/G beads. The TLR4 complex was determined TLR4 and mindin proteins in the complex. The whole cell lysate was used to detect TLR4 and mindin by Western blot

**Fig. 10:**
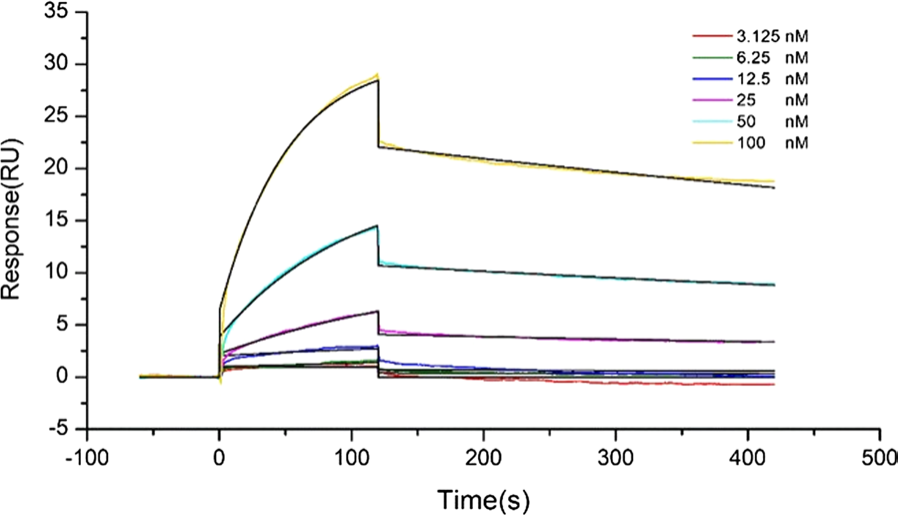
Binding assy of mindin to TLR4 proteins by SPR. Different concen-trations of TLR4 protein (3.125, 6.25, 12.5, 25.0, 50.0, 100.0 nM) were performed to analyse the binding and dissociation rate constants between mindin and TLR4 proteins. The binding constant of them was calculated to evaluate protein binding

The Co-IP assays of mindin and TLR4 proteins were carried out. The mindin and TLR4 proteins of whole cell lysate were both determined in the cells by Western blot (Input). And TLR4 protein was immunoprecipitated from the supernatant using anti-TLR4 antibody followed by agarose beads. Western blot analysis confirmed mindin in the TLR4-bead complex from the IP procedure, as well as TLR4 itself (IP). The result showed that this two proteins were in a complex with each other (Fig. 9). Futhermore, the SPR result showed that there was a strong coupling between mindin protein and TLR4 protein, in which the binding constant of them was 3.25 × 10^–9^ (Fig. 10). In brief, these evidences point out that mindin protein interacts directly with TLR4 protein.

### Mindin was expressed massively in renal tubule tissues of hydronephrosis patients

**Fig. 11:**
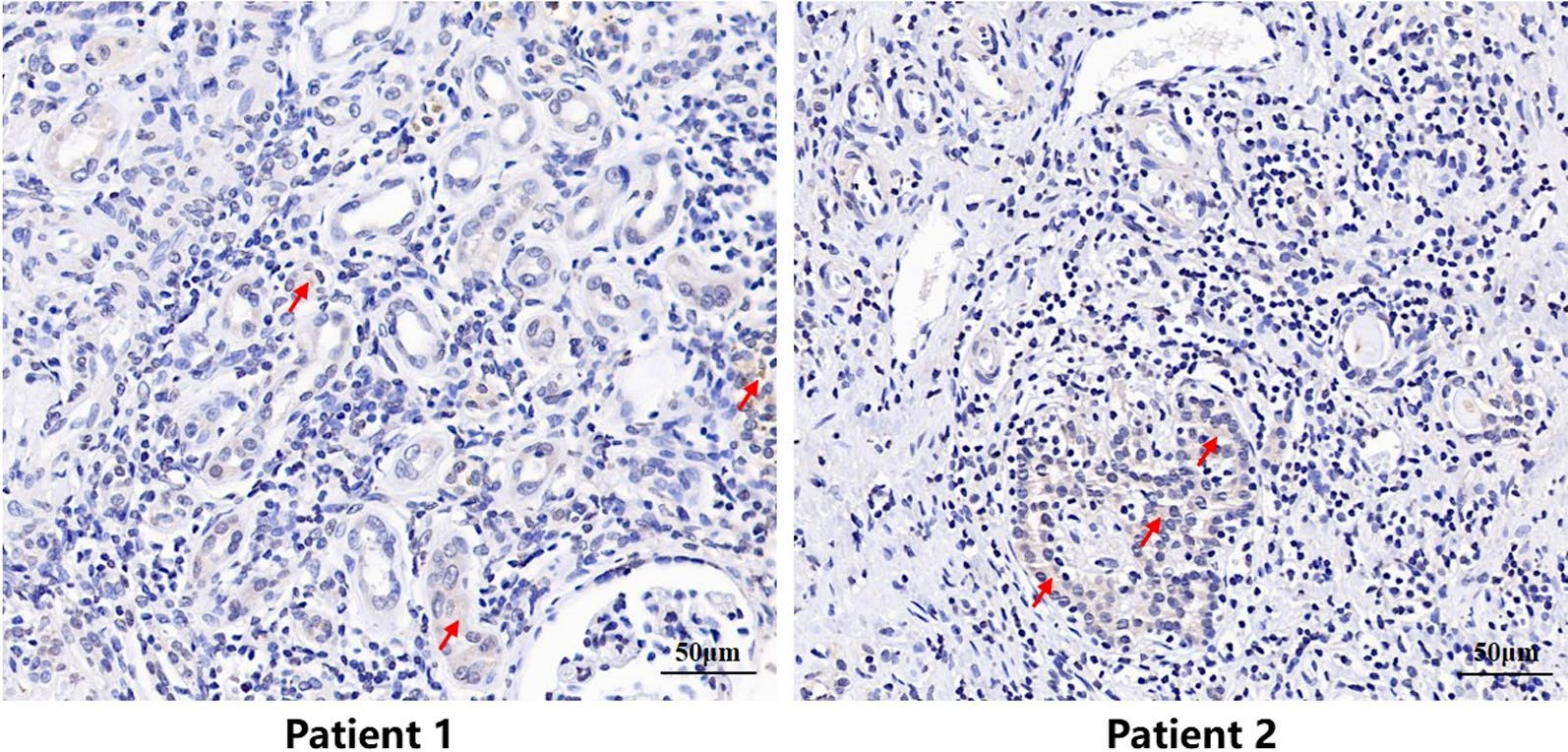
Mindin protein was expressed massively in renal tubule tissues of human hydronephrosis patients by immunohistochemistry. Red arrow indicate mindin expression

The kidney pathological tissues were further examined and studied in human patients. The results showed that mindin was expressed massively in renal tubule tissues of hydronephrosis patients by immunohistochemistry (Fig. 11).

## Discussion

In recent years, the role and function of mindin in different organs have been investigated (Bian et al. 2012; Zhu et al. 2014). Previous studies have reported that mindin was identified as a critical mediator in livers and brains following IR injury (Sun et al. 2015; Wang et al. 2013). And the mindin levels of serum and urine were elevated in diabetic nephropathy (DN) patients and associated with the development of DN (Kahvecioglu et al. 2015; Murakoshi et al. 2011). However, little is known regarding the function of mindin in ischemic AKI progression. In our study, we have demonstrated for the first time that mindin was expressed massively in renal tubule tissues of hydronephrosis patients. And renal IR drastically upregulates mindin expression and that mindin is detected in renal tubules of mice. Moreover, after subjected to IR insults, the kidney tissues showed milder damage in mindin KO mice, while serum BUN and Cre levels were significantly decreased in mindin deficiency mice compared with their WT counterparts. These results implied that the deletion of mindin revealed a renoprotective effect following renal IR.

Many literatures confirmed an important role of neutrophil and macrophage infiltration in the establishment of AKI (Kundert et al. 2018; Raup-Konsavage et al. 2018). These inflammatory cells promote the release of inflammatory mediator, such as inflammatory cytokines, reactive oxygen species, and proteolytic enzymes, which could induce cellular apoptosis and promote further kidney damage (Rabb et al. 2016). Besides, these secreted inflammatory mediators would in turn further induce the accumulation of immunocyte and exacerbate inflammatory cascade (Rabb et al. 2016). Increasing evidence has demonstrated that inhibition of inflammatory response may influence the development of ischemic AKI (Bai et al. 2019). In recent studies, mindin ablation was demonstrated to inhibit IR mediated inflammatory responses in livers and brains and ameliorate organs damage (Sun et al. 2015; Wang et al. 2013). In our work, we found that deleting mindin inhibited neutrophils and macrophages infiltration and the production of TNF-α and MCP-1 in the ischemic kidney of mindin deficiency mice. In addition, the secretion of TNF-α and MCP-1 was dramatically enhanced by mindin overexpression after HR in vitro. These results indicated that mindin deficiency may protects the kidney against IR injury via alleviating inflammatory responses. The extracellular matrix (ECM) proteins, which function as integrin ligands, may participate in inflammatory cell recruitment by interacting with integrins (Li et al. 2009). As an ECM protein, mindin F-spondin (FS) domain mediates integrin binding which is important for inflammatory cell recruitment and T-cell initiation (Li et al. 2009). On the other hand, mindin functions as a pattern-recognition molecule for bacterial and viral pathogens through its thrombospondin-type 1 repeat (TSR) domain (He et al. 2004). Additionally, pattern recognition receptors (PRRs) and integrins play important role in inflammatory cell recruitment and proinflammatory cytokines production after renal ischemic injury (Farrar et al. 2012; Molina et al. 2005). Therefore, we observed milder inflammatory response in mindin KO mice, this probably because PRRs and/or integrins induced inflammatory signaling activation after kidney IR injury has been suppressed.

JNK and NF-κB are common downstream transcriptional factors of TLR4 signaling (Qiu et al. 2019; Kochumon et al. 2018; Lai et al. 2017). Upon ligand recognition, TLR4 can binds and contacts downstream mitogen-activated protein kinases (MAPKs) and NF-κB via receptor dimerization (Liu et al. 2016). Activated MAPKs and components of the NF-κB signaling initiate an inflammation by the release of proinflamm-atory mediators and attraction of inflammatory cells (Qiu et al. 2019). In the context of ischemic AKI, accumulating evidences demonstrate that the TLR4/MAPK/NF-κB pathway plays a crucial role in regulating inflammatory responses during renal IR injury; and inhibiting the TLR4/MAPK/NF-κB signaling can protect kidney from IR injury (Sun et al. 2018). In the present study, we observed that ablation of mindin suppressed the activation of TLR4/JNK/NF-κB signaling after IR injury. The overexpression of mindin promoted the activation of TLR4/JNK/NF-κB signaling after HR in vitro. In contrast, mindin knockdown inhibited the activation of TLR4/JNK/ NF-κB signaling after HR in vitro. Thus, it can be deduced that mindin deficiency reduces renal IR-induced inflammatory responses by suppressing TLR4/JNK/NF-κB signaling pathway.

TLR4 is a transmembrane protein and major PRR that detect motifs on pathogens and host material. In addition to the recognition of pathogen-associated molecular patterns, TLR4 also recognize non microbial endogenous molecules, which has expanded this field beyond sepsis into conditions of autoimmunity and inflammation (Arslan et al. 2010). TLR4 is critical for the attraction of inflammatory cells and the release of proinflammatory factors to further stimulate both the innate and adaptive immune systems (Basile et al. 2012). It is already demonstrated that both proximal and distal tubules constitutively express TLR4, whose expression is upregulated post IR insult (Arslan et al. 2010). The TLR4 plays a proinflammatory and subsequent detrimental role during IR insult contributing to renal dysfunction and tissue damage (Ye et al. 2017). Previous studies using TLR4 knockout mice have demonstrated a protective effect on renal function, chemokine production and inflammatory cellular infiltration in models of renal IR (Rusai et al. 2010; Pulskens et al. 2008). In our study, we found that the expression levels of TLR4 was reduced in mindin KO mice compared with WT mice after renal IR injury. In vitro, higher TLR4 levels were found in mindin overexpression cells compared with control cells after HR.

JNK belongs to the MAPK family, and its function is crucial for signal transduction in both epithelial and endothelial cells. It is established that the JNK pathway serves as a major "on" switch of renal inflammation and cell death in response to a variety of stimuli (Basu and Krishnamurthy 2010). Upon activation of JNK, transcription factors in the cytoplasm or nucleus are, in turn, activated, triggering the expression of target genes causing biological responses (Lai et al. 2017). It can induces activation of the AP-1 transcription factor which promotes expression of several proinflammatory mediators, including TNF-α (Chen et al. 2014). In addition, JNK pathway can promotes renal injury through promoting inflammatory cell recruitment (Amos et al. 2018). Several evidences have demonstrated that JNK signaling pathway mediated renal damage (Kanellis et al. 2010; Wang et al. 2017). Recent study revealed that the levels of both total and phosphorylated JNK were comparable in liver between mindin KO and WT mice following IR injury (Sun et al. 2015). However, as shown in our work, IR insult activated JNK in WT mice, whereas the expression level of phosphorylated JNK was dramatically lower in kidneys from mindin-deficient mice. In contrast, compared with control cells, mindin overexpression TECs exhibited increased phosphorylated of JNK levels in response to HR stimulation. NF-κB, an important inflammatory signaling molecule, modulates the expression levels of adhesion molecules, cytokines, and chemokines in the ischemic AKI (Zhang et al. 2018). IκBα phosphorylation is induced by renal ischemic injury, which promotes nuclear translocation and activation of NF-κB (Kusch et al. 2013). Inflammatory mediators, such as TNF-α and MCP-1, are controlled by NF-κB via the κB-binding motif in the promoter region (Chen et al. 2001). In the present study, we found significantly lower levels of phospho-p65 and phospho-IκBα in kidney tissues from mindin KO mice than in WT mice after IR insult. In contrast, compared with control cells, mindin overexpression TECs exhibited increased phosphorylated P65 and IκBα levels in response to HR stimulation. Collectively, these data demonstrate that the loss of mindin effectively suppressed the activation of TLR4/JNK/NF-κB pathway during renal IR injury.

Various surface molecules were used by microbial pathogens to combine to host ECM components resuting in an effective infection. As a member of the mindin-F-spondin family of secreted ECM protein, mindin was found to be a pattern-recognition molecule which is important in the initiation of innate immune response (He et al. 2004). However, how mindin is involved in the TLR4/JNK/NF-κB signaling pathway is still unknown at present. Therefore, we performed SPR test to demonstrate the binding of mindin to TLR4. The result showed that there was a strong coupling between mindin protein and TLR4 protein, in which the binding constant of them was 3.25 × 10^–9^. Futhermore, the Co-IP assays of mindin and TLR4 were carried out, and the result showed that mindin and TLR4 proteins were in a complex with each other. In brief, these evidences point out that mindin protein interacts directly with TLR4 protein.

In summary, the current study provides the first evidence that mindin is involved in the process of renal IR injury. The knockout of mindin prevents renal IR injury by suppressing inflammatory response. The underlying mechanisms for this effect might be related to the down regulation of TLR4/JNK/NF-κB signaling activation. Whether mindin plays a role in long-term recovery following renal IR needs further investigation. Nevertheless, blockade of mindin has shown to be helpful in reducing renal IR injury and may be useful as a therapeutic option in preventing AKI. Our study limitations was that mindin inhibitors were not used to verify the efficacy in renal IR injury animals, so further experiments would be performed by our group in future.

## Conclusion

This study showed that mindin is a critical modulator of renal IR injury through regulating TLR4/JNK/NF-κB signaling pathway mediated inflammatory responses.

## Data Availability

All data generated or analyzed during this study are included in this published article.

## References

[CR1] Amos LA (2018). ASK1 inhibitor treatment suppresses p38/JNK signalling with reduced kidney inflammation and fibrosis in rat crescentic glomerulonephritis. J Cell Mol Med.

[CR2] Arslan F, Keogh B, McGuirk P, Parker AE (2010). TLR2 and TLR4 in ischemia reperfusion injury. Mediators Inflamm.

[CR3] Bai T (2019). Cryptotanshinone ameliorates renal ischaemia-reperfusion injury by inhibiting apoptosis and inflammatory response. Basic Clin Pharmacol Toxicol.

[CR4] Basile DP, Anderson MD, Sutton TA (2012). Pathophysiology of acute kidney injury. Compr Physiol.

[CR5] Basu A, Krishnamurthy S (2010). Cellular responses to Cisplatin-induced DNA damage. J Nucleic Acids.

[CR6] Bian ZY (2012). Disruption of mindin exacerbates cardiac hypertrophy and fibrosis. J Mol Med (Berl).

[CR7] Cen C (2016). Deficiency of cold-inducible ribonucleic acid-binding protein reduces renal injury after ischemia-reperfusion. Surgery.

[CR8] Chen X (2014). Curcumol exhibits anti-inflammatory properties by interfering with the JNK-mediated AP-1 pathway in lipopolysaccharide-activated RAW264.7 cells. Eur J Pharmacol..

[CR9] Chen F, Castranova V, Shi X (2001). New insights into the role of nuclear factor-kappaB in cell growth regulation. Am J Pathol.

[CR10] Cooper CM, Fenves AZ (2015). Before you call renal: acute kidney injury for hospitalists. J Hosp Med.

[CR11] Farrar CA (2012). Inhibition of TLR2 promotes graft function in a murine model of renal transplant ischemia-reperfusion injury. FASEB J.

[CR12] Feinstein Y, Klar A (2004). The neuronal class 2 TSR proteins F-spondin and Mindin: a small family with divergent biological activities. Int J Biochem Cell Biol.

[CR13] He YW (2004). The extracellular matrix protein mindin is a pattern-recognition molecule for microbial pathogens. Nat Immunol.

[CR15] Jia W, Li H, He YW (2008). Pattern recognition molecule mindin promotes intranasal clearance of influenza viruses. J Immunol.

[CR16] Kanellis J (2010). JNK signalling in human and experimental renal ischaemia/reperfusion injury. Nephrol Dial Transplant.

[CR17] Kahvecioglu S (2015). Evaluation of serum Spondin 2 levels in the different stages of Type 2 diabetic nephropathy. Nephrology (carlton).

[CR18] Kono H (2013). Effect of a novel nuclear factor-kappaB activation inhibitor on renal ischemia-reperfusion injury. Transplantation.

[CR19] Kochumon S (2018). Palmitate activates CCL4 expression in human monocytic cells via TLR4/MyD88 dependent activation of NF-kappaB/MAPK/ PI3K signaling systems. Cell Physiol Biochem.

[CR20] Kundert F (2018). Immune mechanisms in the different phases of acute tubular necrosis. Kidney Res Clin Pract.

[CR21] Kusch A (2013). Novel signalling mechanisms and targets in renal ischaemia and reperfusion injury. Acta Physiol (oxf).

[CR22] Lai JL (2017). Indirubin inhibits LPS-induced inflammation via TLR4 abrogation mediated by the NF-kB and MAPK signaling pathways. Inflammation.

[CR23] Li Y (2009). Structure of the F-spondin domain of mindin, an integrin ligand and pattern recognition molecule. EMBO J.

[CR24] Liu D (2016). Flavonoids from Radix Tetrastigmae inhibit TLR4/MD-2 mediated JNK and NF-kappaB pathway with anti-inflammatory properties. Cytokine.

[CR25] Mizuno S, Nakamura T (2005). Prevention of neutrophil extravasation by hepatocyte growth factor leads to attenuations of tubular apoptosis and renal dysfunction in mouse ischemic kidneys. Am J Pathol.

[CR26] Molina A (2005). Renal ischemia/reperfusion injury: functional tissue preservation by anti-activated {beta}1 integrin therapy. J Am Soc Nephrol.

[CR27] Murakoshi M (2011). Mindin: a novel marker for podocyte injury in diabetic nephropathy. Nephrol Dial Transplant.

[CR28] Perico N, Remuzzi G (2015). Acute kidney injury: more awareness needed, globally. Lancet.

[CR29] Pulskens WP (2008). Toll-like receptor-4 coordinates the innate immune response of the kidney to renal ischemia/reperfusion injury. PLoS ONE.

[CR30] Qiu Y, Wu Y, Zhao H, Sun H, Gao S (2019). Maresin 1 mitigates renal ischemia/reperfusion injury in mice via inhibition of the TLR4/MAPK/NF-kappaB pathways and activation of the Nrf2 pathway. Drug Des Devel Ther.

[CR31] Rabb H (2016). Inflammation in AKI: current understanding, key questions, and knowledge gaps. J Am Soc Nephrol.

[CR32] Raup-Konsavage WM (2018). Neutrophil peptidyl arginine deiminase-4 has a pivotal role in ischemia/reperfusion-induced acute kidney injury. Kidney Int.

[CR33] Rusai K (2010). Toll-like receptors 2 and 4 in renal ischemia/reperfusion injury. Pediatr Nephrol.

[CR34] Song Y (2013). Bis-N-norgliovictin, a small-molecule compound from marine fungus, inhibits LPS-induced inflammation in macrophages and improves survival in sepsis. Eur J Pharmacol.

[CR35] Sun P (2015). Mindin deficiency protects the liver against ischemia/reperfusion injury. J Hepatol.

[CR36] Sun Y, Xun L, Jin G, Shi L (2018). Salidroside protects renal tubular epithelial cells from hypoxia/reoxygenation injury in vitro. J Pharmacol Sci.

[CR37] Tian H (2014). The optimal timing of continuous renal replacement therapy for patients with sepsis-induced acute kidney injury. Int Urol Nephrol.

[CR38] Wang C (2017). Rutaecarpine alleviates renal ischemia reperfusion injury in rats by suppressing the JNK/p38 MAPK signaling pathway and interfering with the oxidative stress response. Mol Med Rep.

[CR39] Wang L (2013). Mindin is a critical mediator of ischemic brain injury in an experimental stroke model. Exp Neurol.

[CR40] Yang Z (2016). Hypothermic machine perfusion increases A20 expression which protects renal cells against ischemia/reperfusion injury by suppressing inflammation, apoptosis and necroptosis. Int J Mol Med.

[CR41] Ye HY (2017). Chlorogenic acid attenuates lipopolysaccharide-induced acute kidney injury by inhibiting TLR4/NF-kappaB signal pathway. Inflammation.

[CR42] Zhang J (2018). Renoprotective effect of erythropoietin via modulation of the STAT6/MAPK/NF-kappaB pathway in ischemia/reperfusion injury after renal transplantation. Int J Mol Med.

[CR43] Zhu LH (2015). Mindin regulates vascular smooth muscle cell phenotype and prevents neointima formation. Clin Sci (lond).

[CR44] Zhu LH (2014). Mindin/Spondin 2 inhibits hepatic steatosis, insulin resistance, and obesity via interaction with peroxisome proliferator-activated receptor alpha in mice. J Hepatol.

